# Awake Sternal Fixation Using the Ultrasound-Guided Superficial Parasternal Intercostal Plane Block in a Patient With Cervical Spine Fracture

**DOI:** 10.7759/cureus.28618

**Published:** 2022-08-31

**Authors:** Burhan Dost, Mehmet G Taflan, Cengiz Kaya, Selcuk Gurz, Serkan Tulgar

**Affiliations:** 1 Department of Anesthesiology and Reanimation, Ondokuz Mayis University, Samsun, TUR; 2 Department of Thoracic Surgery, Ondokuz Mayis University, Samsun, TUR; 3 Department of Anesthesiology and Reanimation, Samsun Training and Research Hospital, Samsun University, Samsun, TUR

**Keywords:** cervical spine fracture, fascial plane blocks, ultrasonography, nerve block, sternum trauma

## Abstract

We present the use of a bi-level, bilateral ultrasound-guided (US-guided) superficial parasternal intercostal plane block (S-PIP) for main anesthetic method in a 71-year-old patient with a C2 vertebral fracture undergoing repair of a sternal fracture. Conscious sedation was provided using midazolam and a remifentanil infusion. The patient had an uneventful recovery and was discharged from the hospital on the first postoperative day without complications. An US-guided S-PIP should be considered when patients are deemed at high risk for general anesthesia, especially in trauma patients with a cervical spine fracture.

## Introduction

Fascial plane blocks have been used in many surgeries in recent years, especially with the widespread use of ultrasound (US) in anesthesia practice. They have been used successfully as the primary anesthetic method in many different surgeries [1,2]. Superficial parasternal intercostal block (S-PIP), formerly known as pecto-intercostal fascial plane block, is a relatively new fascial plane block that provides analgesia in the parasternal area. It has been found to decrease opioid consumption following median sternotomy by anesthetizing the anterior cutaneous branches of the thoracic intercostal nerves (Th2-6). In this block, local anesthetic is injected into the space between the pectoralis major and internal intercostal muscles close to the sternum [3]. Here, we present the successful use of bi-level, bilateral US-guided S-PIP block with conscious sedation for surgical anesthesia in a patient with a cervical spine fracture and scheduled for traumatic sternal fracture surgery.

## Case presentation

**Figure 1:**
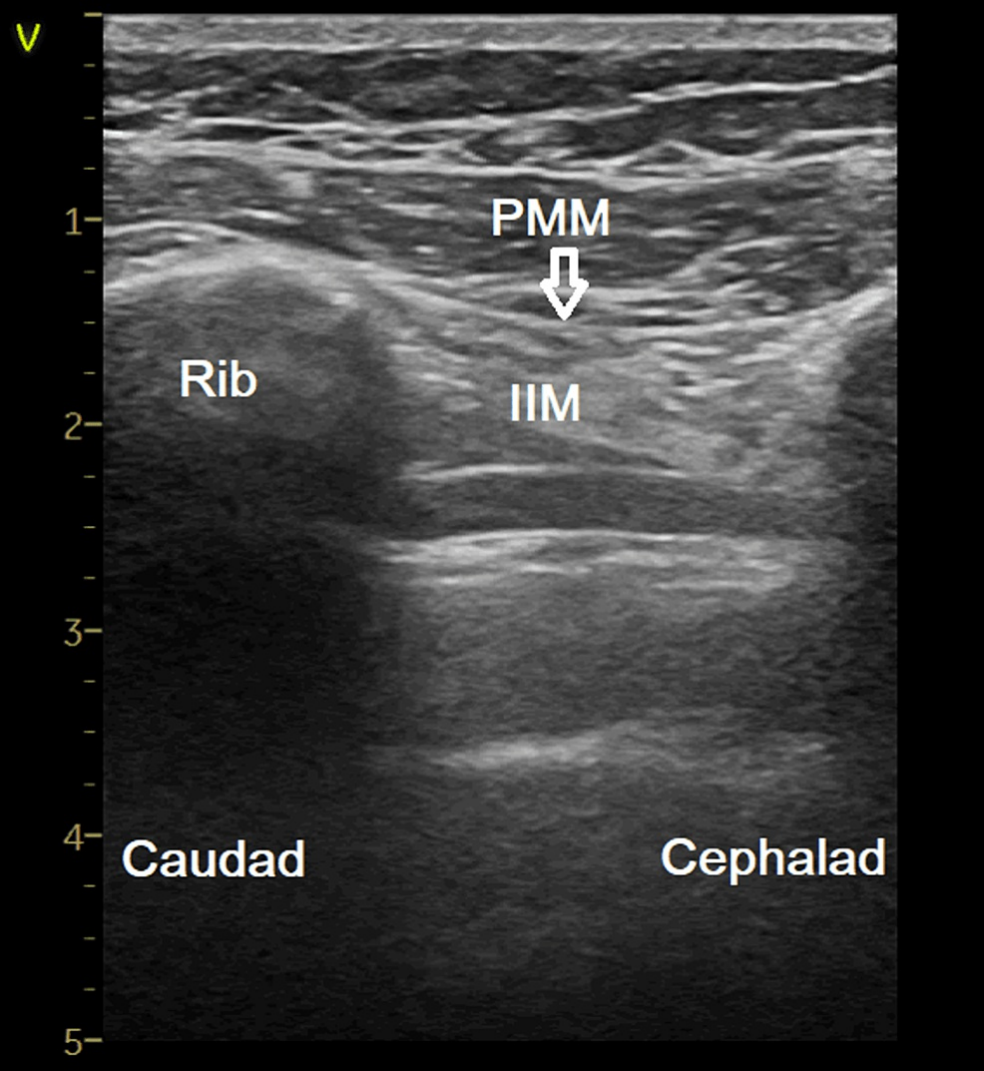
The relevant sonoanatomy for when using an ultrasound-guided superficial parasternal intercostal plane block. The arrow represents the direction of the needle where to inject local anesthetic. PMM: Pectoralis major muscle; IIM: Internal intercostal muscle.

A 71-year-old male (170 cm tall, weighing 80 kg, American Society of Anesthesiologists [ASA] II) presented with a sternum fracture and was scheduled for a sternal fixation. The preoperative assessment indicated diabetes mellitus and coronary artery disease. There was a fragmented fracture in the sternum corpus due to falling from a height one month ago. In addition, there was a compression fracture in the C2 vertebral body. We planned a US‐guided S-PIP block with sedation in this patient due to the disadvantages of general anesthesia, such as difficult airway management and worsening spinal cord injury. With proper preparation for a difficult airway, we could have emergently handled the patient's airway if the block had been insufficient during surgery. The patient was informed about all potential risks, and the patient consented to use his clinical data. After standard ASA monitoring (noninvasive blood pressure, 3-lead electrocardiogram, and oxygen saturation), the patient was started on oxygen (6 L/min flow rate) and received 2 mg of midazolam IV before the administration of the US-guided S-PIP block. Remifentanil infusion was started before the block and continued throughout the procedure. For the patient's communication, remifentanil was titrated at 0.05-0.2 mcg/kg/min infusion to maintain their Ramsay sedation scores at 2 (awake, calm, watching around) and 3 (sleeping, responding to verbal stimuli). Ondansetron 4 mg was administered IV to the patient as a prophylactic for nausea and vomiting, and paracetamol 1 gr IV was given as an adjunct for multimodal analgesia. The patient was positioned supine, and an aseptic technique was used. US guidance was used, as described by Kaya C et al. [3]. In the parasagittal plane, 2-3 cm lateral to the midline, a linear US probe (8-13 MHz, GE LOGIQ V1 US System, USA) was first positioned between the second and third intercostal spaces. Using an in-plane approach, a 22-gauge, 50-mm needle was then advanced from caudal to cranial, targeting the fascial plane between the pectoralis major and internal intercostal muscles (Figure 1).

**Figure 2:**
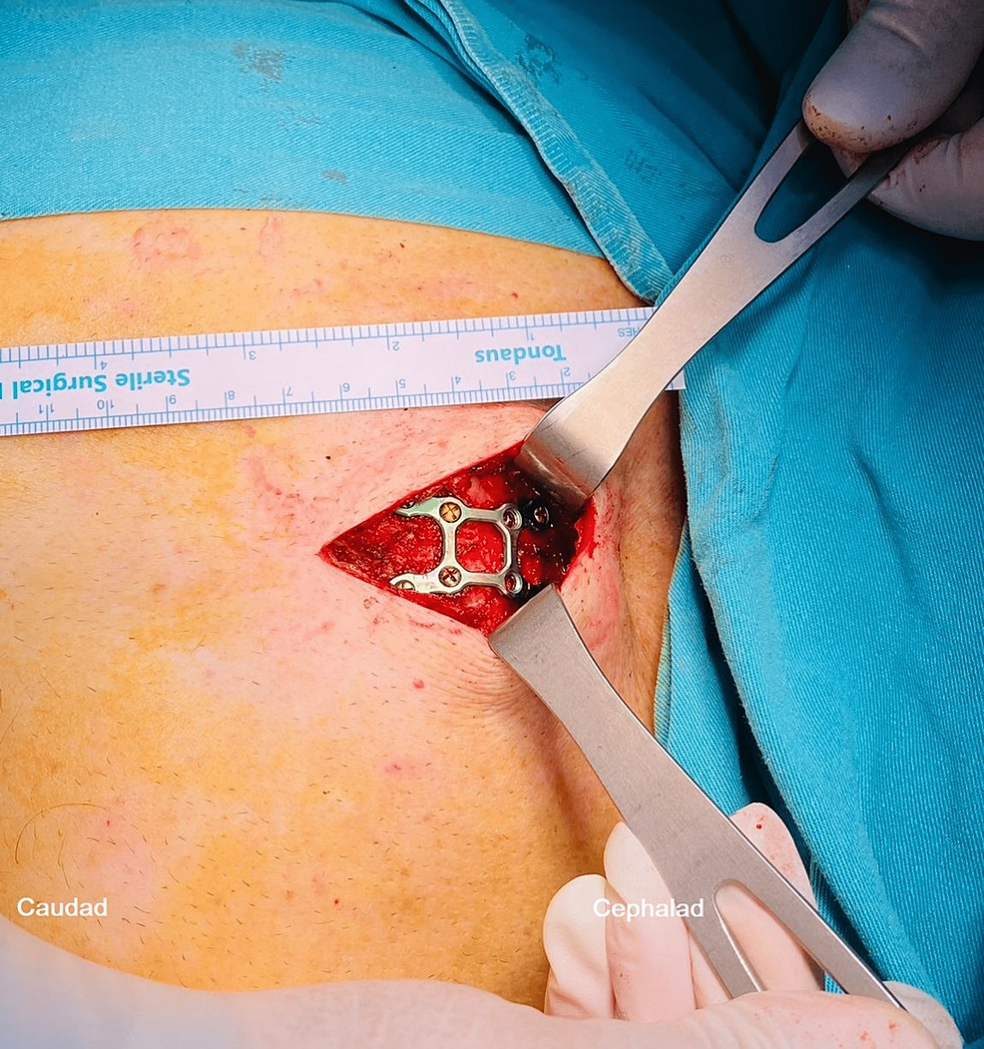
Image shows a surgical incision (approximately 7 cm) and the sternal plate.

Hydro dissection was performed with 1-3 mL of 0.9% normal saline until the distribution was seen in the fascial plane to confirm the needle tip's placement. Next, 15 mL of 0.25% bupivacaine was administered into the interfascial plane following negative aspiration. While the injection was being performed, the distribution of local anesthetic (LA) in the craniocaudal direction was seen in real-time. With 15 mL of 0.25% bupivacaine, the third and fourth intercostal spaces performed the same procedure. Next, an S-PIP block was performed on the other side of the sternum to ensure bilateral blockade. After performing the block, the sensory block (T2-T6 dermatomes) was examined with one pinprick sensation (50-mm long, 22G short bevel; Stimuplex Ultra 360, B. Braun, Germany) every 5 minutes. The anesthetic level required for surgery was accomplished 30 minutes after the S-PIP. Even when the patient remained hemodynamically stable throughout the surgery, the patient complained of mild discomfort during the deep incision and was treated with an increased rate of remifentanil infusion and IV propofol (40 mg total in divided doses). An incision of approximately 7 cm was made, and the connective tissues of the fracture area were dissected. An appropriate sternal plate was placed on the fracture site and fixed to the sternum with 10 mm drilling screws (Figure 2).

The surgery was completed in 90 minutes without the surgeon's local supplementation. The patient received acetaminophen (1 gr) every eight h/24 hours. In the postoperative follow-up, he had not reported any pain for 24 hours. The patient had an uneventful recovery and was discharged from the hospital on the first postoperative day without complications.

## Discussion

Our case demonstrates that sternum surgery can be performed without general anesthesia, even in a patient with a traumatic sternal fracture, by implementing a bi-level, bilateral US-guided S-PIP block under sedation. US-guided S-PIP block was used for rescue pain management of traumatic sternal fracture [4] and rib cage pain in ICU patients [5]. Karapinar YE et al. [6] have recently reported awake sternum revision with US-guided transverse thoracic muscle plane block, which is similar to our case. In addition, awake cardiac surgery using the novel pectoralis‐intercostal‐rectus sheath plane block was reported [7]. However, a survey study involving 119 anesthesia providers reported a lack of experience handling difficult and surgical airways [8]. Considering the importance of stabilization in cervical trauma and the complications of intubation maneuvers in general anesthesia, it would be more reasonable to perform the surgery with a regional technique while awake. In our case, a single injection may cover the dermatomes required for the surgical procedure. However, we preferred the bilateral, bi-level injection technique to increase the local anesthetic spread and used 60 mL of 0.25% bupivacaine. In addition, although our patient had no pain for the first 24 hours, a catheter could have been inserted for more extended pain control, as previously reported [9].

## Conclusions

In conclusion, US-guided S-PIP block should be considered when patients are deemed at high risk for general anesthesia, especially in trauma patients with a cervical spine fracture.

## References

[1] Ahiskalioglu A, Tulgar S, Celik M, Ozer Z, Alici HA, Aydin ME (2020). Lumbar erector spinae plane block as a main anesthetic method for hip surgery in high risk elderly patients: initial experience with a magnetic resonance imaging. Eurasian J Med.

[2] Kaya C, Dost B, Tulgar S (2021). Sacral erector spinae plane block provides surgical anesthesia in ambulatory anorectal surgery: two case reports. Cureus.

[3] Kaya C, Dost B, Dokmeci O, Yucel SM, Karakaya D (2022). Comparison of ultrasound-guided pecto-intercostal fascial block and transversus thoracic muscle plane block for acute poststernotomy pain management after cardiac surgery: a prospective, randomized, double-blind pilot study. J Cardiothorac Vasc Anesth.

[4] Hsu M, Kinthala S, Huang J, Kapoor N, Saththasivam P, Porter B (2022). Pectointercostal fascial plane block for rescue pain management of traumatic sternal fracture following inadequate thoracic epidural block: a case report. J Surg Case Rep.

[5] López-Matamala B, Fajardo M, Estébanez-Montiel B, Blancas R, Alfaro P, Chana M (2014). A new thoracic interfascial plane block as anesthesia for difficult weaning due to ribcage pain in critically ill patients. Med Intensiva.

[6] Karapinar YE, Medetoglu EN, Ozkal MS, Aydin ME, Ahiskalioglu A (2022). Ultrasound-guided transverse thoracic muscle plane block for awake sternum revision in a post-COVID patient. J Anesthesiol Reanim Spec Soc.

[7] Toscano A, Balzani E, Capuano P (2022). Awake cardiac surgery using the novel pectoralis-intercostal-rectus sheath (PIRS) plane block and subxiphoid approach. J Card Surg.

[8] Fayed M, Nowak K, Angappan S, Patel N, Abdulkarim F, Penning DH, Chhina AK (2022). Emergent surgical airway skills: time to re-evaluate the competencies. Cureus.

[9] Raza I, Narayanan M, Venkataraju A, Ciocarlan A (2016). Bilateral subpectoral interfascial plane catheters for analgesia for sternal fractures: a case report. Reg Anesth Pain Med.

